# Pancreatic Neuroendocrine Neoplasms Larger than 4 cm: A Retrospective Observational Study of Surgery, Histology, and Outcome

**DOI:** 10.3390/jcm12051840

**Published:** 2023-02-25

**Authors:** Anna Caterina Milanetto, Anna-Lea Gais Zürcher, Alina David, Matteo Fassan, Claudio Pasquali

**Affiliations:** 1 Pancreatic and Endocrine Digestive Surgical Group, Department of Surgery, Oncology and Gastroenterology, Università degli Studi di Padova, Via Giustiniani 2, 35128 Padova, Italy; 2 Surgical Pathology Unit, Department of Medicine, Università degli Studi di Padova, Via Giustiniani 2, 35128 Padova, Italy

**Keywords:** pancreas, neuroendocrine neoplasm, pancreatic surgery, tumor size

## Abstract

Background: Pancreatic neuroendocrine neoplasms (pNENs) are often detected as large primary lesions, even with distant metastases, and their prognosis may be difficult to predict. Methods: In this retrospective study, we retrieved data of patients treated for a large pNEN in our Surgical Unit (1979–2017) to evaluate the possible prognostic role of clinic-pathological features and surgery. Cox-proportional hazard regression models were used to find possible associations among some variables (clinical features, surgery, and histology) and survival at univariate and multivariate analyses. Results: Among 333 pNENs, we identified 64 patients (19%) with a lesion > 4 cm. Patients’ median age was 61 years, median tumor size was 6.0 cm, and 35 (55%) patients had distant metastases at diagnosis. There were 50 (78%) nonfunctioning pNENs, and 31 tumors localized in the body/tail region of the pancreas. Overall, 36 patients underwent a standard pancreatic resection (with 13 associated liver resection/ablation). Regarding histology, 67% of pNENs were N1, and 34% were grade 2. After a median follow-up of 48 months (up to 33 years), 42 patients died of disease. Median survival after surgery was 79 months, and six patients experienced recurrence (median DFS 94 months). At multivariate analysis, distant metastases were associated with a worse outcome, while having undergone radical tumor resection was a protective factor. Conclusions: In our experience, about 20% of pNENs have a size > 4 cm, 78% are nonfunctioning, and 55% show distant metastases at diagnosis. Nevertheless, a long-term survival of more than five years may be achieved after surgery.

## 1. Introduction

Pancreatic neuroendocrine neoplasms (pNENs) are rare tumors and account for approximately 5% of all pancreatic tumors [1]. According to the SEER database (2000–2012), the annual incidence of pNENs is increasing to 0.48 per 100,000 persons [2]. An estimated 70 to 80% of pNENs are non-functioning (NF), and up to 60% of patients may present with metastatic disease at diagnosis [1,3,4]. Due to the lack of specific/endocrine symptoms at presentation, patients are often incidentally diagnosed with a pNEN, and tumor size is one of the first tumor characteristics to be described. In the European Neuroendocrine Tumor Society (ENETS) TNM classification currently used for pNENs [5], tumor size is considered for T parameter evaluation, and TNM stage is one of the tumor features determining the prognosis of pNENs. Median overall survival (OS) may be 3.6 years for all pNEN stages, with a range from 20 months (1.7 years) in patients with distant metastases to 230 months (19 years) in patients with only pancreatic involvement [2]. Pancreatic NENs are heterogeneous neoplasms with variable malignant potential, and in addition to TNM stage, other parameters which may affect long-term survival have been identified (i.e., neuroinvasion or infiltrative pattern [6], cell differentiation, and grade [7]). The assessment of these features would be of paramount importance for surgical decisions; unfortunately, histological/immunohistochemical results of a preoperative biopsy may not be reliable due to the wide intratumoral biological heterogeneity, especially for large tumors, with a concordance rate of the World Health Organization (WHO) classification between endoscopic ultrasound (EUS)-fine needle aspiration (FNA) and surgical specimens reported to be 57% in tumors > 2 cm in size [8].

The surgical choice is often based on morphological rather than biological findings, and tumor size is considered in the surgical decision making of pNENs. According to the ENETS guidelines, patients with a functioning pNEN or with a pNEN > 2 cm in size should undergo surgery, whereas the others may be included in an active surveillance program [9]. It has been shown that pNENs < 2 cm carry a similar risk of malignancy and a similar survival time to large tumors [10]. Since other studies have failed to identify a correlation between tumor size and prognosis [11,12,13,14], no size cut-off exists beyond which malignancy can be excluded safely [15,16].

Surgery represents the treatment of choice to achieve cure in fit-for-surgery patients, even in the presence of distant metastases. According to the SEER database (2000–2012), survival has improved over time, and patients with a G1-G2 pancreatic neuroendocrine tumor (pNET) with distant metastases showed an OS of 60 months [2]. Regarding pNENs larger than 4 cm, there is a generally held belief that they have a great potential for aggressive behavior [16], and a survival analysis of more than 200 operated patients highlighted that in the absence of nodal and distant metastases, tumor size > 4 cm has an independent prognostic significance [17]. At the same time, it is also clear that even among large tumors, there is substantial biologic variability [16]. 

We retrospectively collected from a 40-year monocentric experience of pancreatic and digestive neuroendocrine surgery a series of patients diagnosed with a pNEN > 4 cm, as described from the initial imaging studies and/or final histology. The aim of the present study was to evaluate the possible prognostic role of clinical presentation, histology, and surgery in those patients.

## 2. Patients and Methods

In this retrospective study, patients with a functioning or nonfunctioning, well or poorly differentiated pancreatic NEN with size larger than 4 cm diagnosed in our Pancreatic and Digestive Endocrine Surgical Unit between January 1979 and December 2017 were enrolled. Records of those patients concerning clinical presentation, surgery or other treatments, and histology were retrieved and retrospectively evaluated from clinical charts. In nonoperated patients, diagnosis of pNEN was established by clinical presentation (for functioning NEN), imaging studies (CT scan, MRI, 111In-somatostatin receptor Scintigraphy, and/or 68Ga-peptide PET/CT), and/or a pancreatic/liver biopsy. Follow-up (FU) closed in December 2019, and only patients with at least two years of follow-up were included. Patients diagnosed with a pNEN > 4 cm after December 2017 and having less than two years of follow-up were excluded. Overall survival (OS) and disease-free survival (DFS) were defined either by using a personal telephone interview or at the last FU visit, which included imaging studies (CT scan, MRI and/or 68Ga-peptide positron emission tomography (PET/CT). The study protocol was approved by the local Ethics Committee (project code 2872p).

The following data were analyzed: age, gender, medical history (MEN-1 syndrome, functioning/NF-pNENs, incidental pNENs defined as tumors in asymptomatic patients who underwent abdominal imaging studies for unrelated causes), (preoperative) TNM stage at imaging studies, tumor localization in the pancreas, type of surgery (enucleation, pancreatico-duodenectomy, distal pancreatectomy, total pancreatectomy, associated liver surgery), operative time (min), blood loss (mL), post-operative complications, hospital stay (days), overall morbidity, early post-operative mortality (within 90 days from surgery), reoperation rate, post-pancreatectomy hemorrhage [18], and post-operative pancreatic fistula [19]. Known factors influencing the complication rate may be the diameter of the pancreatic duct, the pancreatic texture, the anastomosis technique, and the type of suture used [20].

The diagnosis and grading of pNENs were carried out according to the WHO 2019 Classification of Digestive System Tumours [7] and the ENETS TNM classification [5]. We evaluated tumor size (defined either by the largest diameter on CT scan/MRI in nonoperated patients, or by the largest diameter at final histology for operated patients), lymph node (LN) metastases (Nx, N0, N1), distant metastases (M0, M1), and tumor grade (G1, G2, and G3, assessed by Ki67 labelling index). In the cases diagnosed before 1998, we could only distinguish between pNET and pancreatic neuroendocrine carcinoma (pNEC), according to cell differentiation. Immunohistochemical analysis was performed for synaptophysin, chromogranin, neuron-specific enolase, and for expression of other hormones (insulin, glucagon, somatostatin, pancreatic polypeptide, and gastrin, where applicable). A mixed neuroendocrine-non-neuroendocrine neoplasm (MiNEN) was diagnosed when both neuroendocrine and non-neuroendocrine components exceeded 30% within a neoplasm [21]. 

Overall survival (OS) was defined as the time from surgery (or from diagnosis in those nonoperated patients) to the death of the patient, or to the end of follow-up (FU) for censored patients. Disease-free survival (DFS) was defined as the time from radical surgical resection to post-operative tumor recurrence at least six months after surgery. 

## 3. Statistical Analysis

Survival curves for OS were estimated using the Kaplan–Meier method. We evaluated the *p*-value of non-parametric rank test optimized for censored data (a generalized two-sample Wilcoxon test). Cox-proportional hazard regression models were used to evaluate the possible association among some variables (patients’ characteristics, tumor features, and treatments) and survival at univariate and multivariate analyses. A *p* value less than 0.05 was considered statistically significant. Statistical analysis was performed using the R program (packages “Survminer” and “Survival”, version 3.6.3) [22].

## 4. Results

Among 333 patients diagnosed with a pNEN in our Pancreatic and Digestive Endocrine Surgical Unit, we identified 64 (19.2%) patients with a pNEN > 4 cm, with a median age of 61 (range, 26–79) years (Table 1). 

Median tumor size was 6.0 (range, 4.0–15.0) cm, and 50 patients presented with a NF-NEN (19 of them as an incidental finding). Overall, 31 patients showed a lesion localized at the body-tail region of the pancreas, 34 patients presented with liver metastases, and 15 of them had also metastases in other distant organs. In addition, 36 patients underwent surgery of the primary pNEN (21 distal pancreatectomies, 10 pancreatoduodenectomies, 2 total pancreatectomies, 2 enucleations with regional LN dissection, and 1 distal pancreatectomy associated to pancreato-duodenectomy in MEN-1) (Table 2). Notably, the palliative resection group included 15 patients who underwent surgery of the primary pNEN in the presence of liver metastases, and 6 of them also had metastases in other distant organs. In 13 patients, an associated surgical/ablative treatment of liver metastases was performed, and 6 patients also underwent trans-arterial embolization (TAE) of liver metastases in the early post-operative time. 

Post-operative surgical complications occurred in 14 patients (morbidity rate 39%), and all but 4 cases were managed conservatively (reoperation rate 11%). One patient died of liver/renal failure post-operatively (mortality rate 2.8%). The other 28 patients were not operated because of locally advanced disease invading adjacent organs and/or major blood vessels, wide systemic spread of the disease, or age/comorbidities. Particularly, seven patients with functioning pNENs who were not operated received systemic and loco-regional treatment (i.e., chemotherapy, somatostatin analogues, everolimus, and/or liver microwave ablation). Thirty-five patients had a stage IV disease, and the majority of patients had a pNET (47 out of 57). Among 41 available tumor grade, 32 pNENs were classified as grade 1–2 (median Ki67 7%, range 0–70%). Since 1993, 111-In Scintigraphy, 18F-FDG PET/CT, and then 68Ga-peptide PET/CT have been performed in our center, and we evaluated the concordance between functional imaging and histological findings. Scintigraphy/Ga-PET showed a high sensitivity for pNETs (21 out of 25 G1-G3 pNETs had a positive imaging), and FDG-PET had the highest sensitivity for pNECs (all six NEC/G3 MiNENs had a positive imaging). Both Scintigraphy and FDG-PET had a low specificity: four out of six pNECs were Scintigraphy positive, and 18 out of 24 G1-G3 pNETs were FDG-PET positive.

Data on FU and survival are reported in Table 1. Considering together operated and nonoperated patients, 5-year OS and 10-year OS were 47% and 30%, respectively. After a median FU of 48 months (range, 0.3–395), 22 patients were alive (or died from other causes). Forty-two patients died of disease after a median survival of 21 months (range, 0.3–160). Regarding the 36 operated patients, median OS was 79 months (range 3.8–395), and 19 of them (53%) died of disease. Twenty-one operated patients (58% of the operated group, 33% of all patients) who presented with a M0 pNEN at diagnosis, and who were operated with curative intent, achieved a median survival of 126 months (range, 16–395). After a median DFS of 94 months (range, 16–395), six of them experienced tumor recurrence (five were NF-NENs) and were treated with somatostatin analogues, TAE and/or chemotherapy. Fifteen patients who underwent a palliative resection reached a median survival of 26 months (range, 4–158), which is still longer than that of nonoperated patients (median 18.5 months).

At univariate analysis (Table 3), tumor size did not significantly affect OS (*p* = 0.474), and there was a statistically significant difference in OS for the different age categories (patients’ age divided by quartiles, *p* = 0.035). Regarding surgical operation, the operated patients showed a significantly better prognosis than nonoperated patients (median OS 100 months vs. 24 months, *p* = 0.0013), and patients resected after 1998 did not have a significant survival advantage over those operated before 1998 (*p* = 0.39). 

**Figure 1:**
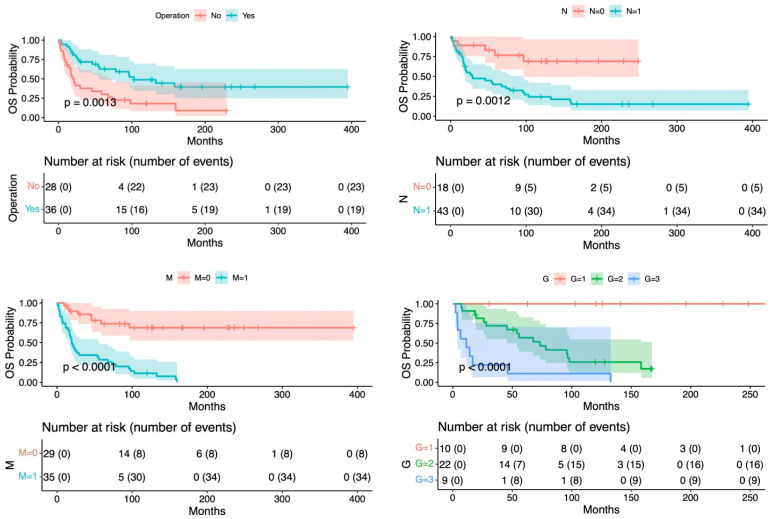
Overall survival related to surgical operation, lymph node metastases, distant metastases, and tumor grade.

As showed in Figure 1, surgery, lymph node status, distant metastases, and tumor grade showed a significant effect on OS. The same variables had a similar effect on OS when considering only the operated patients. 

At multivariate analysis, only age (quartile ≥ 47.5–61 years) as a risk factor (*p* = 0.007), and surgery as a protective factor (*p* = 0.037) were significantly related to the risk of death. Bivariate analysis highlighted distant metastases as a confounding factor with age; thus, the reduced probability of survival may be due to widespread disease rather than to old age.

## 5. Discussion

In our study, we evaluated a single center experience of the treatment of pNENs with size > 4 cm, with 64 patients enrolled among all the pNENs treated in our Surgical Unit in the last 40 years. Final survival analyses showed a statistically significant association between prognosis and LN metastases, distant metastases, tumor grade, and surgical treatment, but tumor size did not affect survival. At multivariate analysis, the presence of distant metastases was associated with a worse outcome, while having undergone radical tumor resection was a protective factor.

In our experience, pNENs with size > 4 cm represent almost 20% of all the pNENs observed and treated (median patients’ age of 61 years), with a median tumor size of 6.0 (up to 15) cm, as reported in a recent series from the SEER database, with 30% of pNENs > 4 cm and a median patients’ age of 58 years [23]. Given their indolent natural history, especially NF-pNENs may reach a huge size before being diagnosed. In fact, 78% of patients presented with a NF-NEN, and 38% of them were completely asymptomatic, as previously shown in a recent study of large NF-pNETs with a similar rate (45.5% of asymptomatic patients) [10]. At univariate analysis, patients with a functioning/symptomatic pNEN did not show a significantly better prognosis when compared to the NF ones. In our experience, the incidence of pNENs > 4cm did not decrease in the last decades, with 69% of pNENs diagnosed after 1998 (almost all having a NF-NEN); this may be due to the high availability of cross-sectional imaging, and to the high sensitivity of some recently introduced techniques (i.e., 68Ga-PET/CT, EUS). Large pNENs were equally located between the head and the body–tail region of the pancreas, whereas in a series by Zhang et al. [23], 44% of tumors were located in the tail of the pancreas. 

Scarpa et al. found that tumor size > 4 cm had an independent prognostic significance at multivariable analysis, in the absence of LN/distant metastases [17], whereas in our series, survival analysis proved that tumor size did not have a statistically significant association with the patients’ prognosis, as previously reported by several other authors [24,25,26,27,28,29,30,31]. Among pNENs with size > 4 cm, the majority were well-differentiated (82.5%) and grade 1–2 (78%) tumors (with a median Ki67 of 7%). Large G1 pNENs were 25%, whereas the rate of G3 tumors was lower (22%) than the 33% reported in a series of pNENs > 4 cm by Cherefant et al. [32]. No correlation was found between Ki67 index and tumor size, confirming the findings of Hamilton et al. [33]. At univariate analysis, a low tumor grade (G1-G2) was significantly related to a long survival (*p* < 0.0001), irrespective of surgery, but the low number of tumor grades available does not allow us to draw further conclusions. In other series of large pNENs, tumor grade and distant metastases have been shown to play a significant role for patients’ survival [23,24,25], being predictors of prognosis independently of tumor size [26,34]. 

Some authors consider tumor size a predictor of LN involvement [24], and others showed that tumor size is significantly related to tumor grade [10]; in both studies, [10,24] tumor size was not related to distant metastases. Recently, Xu et al. [35] showed that both large tumor size (>4 cm) and tumor extending beyond the pancreas (T3-T4) were significantly and independently related to LN metastases. In our series, patients presented mostly with LN metastases (70.5% vs. 42% previously reported in another series of NF-pNETs > 4 cm [32]), and survival of N1 patients was significantly shorter than that of N0 patients (36 vs. 100 months, *p* = 0.0012), irrespective of surgery, whereas Zhang et al. reported no prognostic influence of LN metastases for patients with a pNEN > 4 cm [23]. 

In a review of 9821 pNENs by Bilimoria et al. [36], tumor size > 4 cm was independently associated with a low likelihood of undergoing surgical resection, after excluding for metastatic disease. Surgical resection is the only potentially curative treatment for pNENs, and in our study, surgery was a statistically significant protective factor at multivariate analysis (*p* = 0.0013). Even in the presence of other malignant features, surgery can increase patients’ survival, since 42% of operated patients presented with distant metastases, showing a G2 and a G3 pNET in 50% and 19% of cases, respectively. Even in the subgroup of elderly patients, the protective effect of surgery prevailed. In previous studies, tumor grade [37] and ENETS stage were the only independent prognostic factors of DFS [38], regardless of other clinic-pathologic features. In a multicentric series by Genç et al., pNENs > 4 cm were independently associated with recurrence [39], as shown by other authors [33,40,41,42]. Among 21 M0 operated patients, we observed six recurrences to local/distant LNs and to distant organs (liver and bone) after a median DFS of 94 months; although a relatively long DFS, the low number of recurrences did not allow us to perform further analyses.

In our study, patients presenting with distant (liver) metastases showed a significantly shorter survival when compared to M0 patients (24 vs. 100 months, *p* < 0.0001). Nevertheless, after a palliative resection, M1 patients reached a median OS eight months longer than that of nonoperated patients. From the analysis of SEER database, Hüttner et al. [43] reported that stage IV pNEN patients (n = 75) undergoing palliative primary tumor resection had a significant benefit in both OS and cancer-specific survival, as previously reported in other studies [44,45,46] with a 5-year OS exceeding 60%. In our series, in the same surgical session, 87% of M1 patients received an associated liver surgery and/or ablation, with surgical morbidity and hospital stay similar to patients undergoing a pancreatic resection only. The resection and/or ablation of liver metastases may improve 5-year OS from 25% to 72% compared to nonoperated patients [47]; from the analysis of more than 2000 pNENs of the SEER database, Franko et al. reported the greatest survival benefit for patients who underwent resection of both primary pNEN and liver metastases (*p* < 0.001) [24]. Therefore, our study confirmed previously reported data of an association between survival and surgical treatment among selected patients with both localized and metastatic disease.

Our study has several limitations. First, it is a retrospective study including relatively small-sized subgroups of patients for each treatment procedure described. In addition, the histological criteria for tumor differentiation and grade of pNEN have only been standardized in the last 20 years, and this study covers a period of almost 40 years; therefore, there is some variability in the pathology reports over time (i.e., Ki67 was not included in the routinary pathology reports up to 1998). Nevertheless, we collected a series of 64 patients with a pNEN > 4 cm, observed and treated in the same surgical center with the same standardized approach. Then, the long observation time (median FU 48 months, up to 33 years) is long enough to evaluate the results of surgery for these slow-growing neoplasms, which may give a disease recurrence even after a median time of 94 months (almost eight years). The size of the reported series should not be underestimated; being rare neoplasms, it may be difficult to reach an adequate number of patients and with a sufficiently long FU to obtain statistically significant results. Finally, in this surgical series, we also described data of nonoperated patients, and thus the natural history of the disease may be better understood. 

## 6. Conclusions

Despite imaging studies facilitating the detection of predominantly small lesions, pNENs may reach a considerable size before becoming symptomatic. In the subset of pNENs > 4 cm, tumor size alone is not a prognostic factor, and distant metastases, tumor grade, and LN status are associated with a worse prognosis. Even with a cytoreductive intent in selected patients, surgery can effectively improve survival as independent prognostic factor, and its protective effect prevailed also for elderly and/or M1 patients. Surgery should be the standard treatment for large pNENs, and in the decision-making process for these patients, a surgical evaluation is strongly recommended.

## Figures and Tables

**Table 1:** Clinical history, pathological findings, and follow-up of operated (n = 36) and nonoperated (n = 28) patients with a pancreatic NEN > 4 cm.

		Operated Patients (n = 36)	Nonoperated Patients (n = 28)	Overall (n = 64)
Gender	Male, n Female, n	24 12	12 16	36 28
Age	median (range), years	61.5 (26–78)	60 (38–79)	61 (26–79)
Nonfunctioning, n	Incidental, n Symptomatic, n	13 16	6 15	19 31
Functioning, n	Type, n	7 (3 insulin, 2 glucagon, 2 ZES)	7 (1 insulin, 3 ZES, 1 insulin/ZES, 2 carcinoid)	14
MEN-1 syndrome	No, n Yes, n	29 7	28	57 7
Tumor size ^a^	median (range), cm	6.5 (4.1–15)	6.3 (4.1–11)	6.0 (4.1–15)
Tumor location	Head, n Body-tail, n Diffuse, n	12 21 3	17 10 1	29 31 4
Distant metastases at diagnosis	No, n Liver, n Other ^b^, n	21 15 6	9 19 9	30 34 15
TNM stage ^c^	IIb IIIa IIIb IV	10 1 10 15	4 2 2 20	14 3 12 35
T (n.a. = 1)	3 4	24 12	12 15	36 27
N (n.a. = 3)	0 1	11 25	7 18	18 43
M	0 1	21 15	8 20	29 35
Histology (n.a. = 7)	NET, n NEC, n MiNEN, n	30 5	17 3 2	47 8 2
Grade (n.a. = 23)	1 2 3	9 14 5	1 8 4	10 22 9
Follow-up	median (range), months	79 (3.8–395)	18.5 (0.3–229)	48 (0.3–395)
Status	ANED AWD DOC DOD POD	7 4 6 18 1	- 1 4 23 -	7 5 10 41 1

^a^ tumor size: pathological size for operated patients, radiological size for nonoperated patients. ^b^ in operated patients: adrenal gland, spleen, peritoneum, lung, bone; in nonoperated patients: peritoneum, lung, bone, mediastinum, neck lymph nodes. ^c^ pTNM, pathological TNM for operated patients. cTNM, clinical TNM for nonoperated patients. NEN, neuroendocrine neoplasm. ZES, Zollinger-Ellison syndrome. MEN-1, multiple endocrine neoplasia type 1. NET, neuroendocrine tumor. NEC, neuroendocrine carcinoma. MiNEN, mixed neuroendocrine-nonneuroendocrine neoplasm. ANED, alive and no evidence of disease. AWD, alive with disease. DOC, died of other cause. DOD, died of disease. POD, post-operative death. n.a., not applicable.

**Table 2:** Type of surgery and post-operative outcome of the operated patients (n = 36) with radical (n = 21) or palliative (n = 15) intent.

	Radical Resection (n = 21)	Palliative Resection (n = 15)	Overall (n = 36)
Type of surgery Distal pancreatectomy Pancreatico-duodenectomy Total pancreatectomy Enucleation DP and PD	9 7 2 2 1	12 3	21 10 2 2 1
Associated liver treatment Surgery Ablation ^a^ TAE (postoperatively)	- -	13 11 5 6	13 11 5 6
Operation time: median (range), min	330 (130–550)	270 (205–630)	320 (130–630)
Intraoperative blood loss: median (range), mL	600 (150–2300)	1100 (150–3000)	700 (150–3000)
Hospital stay: median (range), days	14 (9–47)	14.5 (9–34)	14 (9–47)
Surgical morbidity ^b^			14
BL	1	-	1
POPF (grade B)	3	2	5
PPH	1	2	3
Other ^c^	5	-	5
Reoperation	3	1	4
90 day-postoperative mortality	-	1	1

^a^ Alcohol, radiofrequency, and/or microwave. ^b^ Fourteen complications occurred in fourteen patients. ^c^ Two bowel obstruction, two pylorus anastomosis dehiscence, one gastric perforation. DP, distal pancreatectomy. PD, pancreato-duodenectomy. TAE, trans-arterial embolization. BL, biochemical leak. POPF, post-operative pancreatic fistula. PPH, post-pancreatectomy hemorrhage.

**Table 3:** Distribution of the predictor variables and results of univariate analysis for overall survival.

Predictor Variable	Categories (n)	*p*-Value
Gender	male (36) vs. female (28)	0.061
Age, years	<47.5 (16) vs. ≥47.5–61 (15) vs. ≥61–69 (16) vs. ≥69 (17)	0.035
Tumor size, cm	4.1–6.0 (34) vs. >6.0 (30)	0.474
Symptomatic nonfunctioning tumor	yes (31) vs. no (19)	0.37
Operation	yes (36) vs. no (28)	0.0013
Lymph node status (all patients)	N0 (18) vs. N1 (43)	0.0012
Lymph node status (operated patients)	N0 (11) vs. N1 (25)	0.0032
Distant metastases (all patients)	M0 (29) vs. M1 (35)	<0.0001
Distant metastases (operated patients)	M0 (21) vs. M1 (15)	<0.0001
Grade (all patients)	G1 (10) vs. G2 (22) vs. G3 (9)	<0.0001
Grade (operated patients)	G1 (9) vs. G2 (14) vs. G3 (5)	<0.0001

## Data Availability

The data presented in this study are available on request from the corresponding author. The data are not publicly available due to ethical restrictions.

## References

[1] Yao J.C., Hassan M., Phan A., Dagohoy C., Leary C., Mares J.E., Abdalla E.K., Fleming J.B., Vauthey J.-N., Rashid A. (2008). One hundred years after «carcinoid»: Epidemiology of and prognostic factors for neuroendocrine tumors in 35,825 cases in the United States. J. Clin. Oncol..

[2] Dasari A., Shen C., Halperin D., Zhao B., Zhou S., Xu Y., Shih T., Yao J.C. (2017). Trends in the Incidence, Prevalence, and Survival Outcomes in Patients with Neuroendocrine Tumors in the United States. JAMA Oncol..

[3] Metz D.C., Jensen R.T. (2008). Gastrointestinal neuroendocrine tumors: Pancreatic endocrine tumors. Gastroenterology.

[4] Halfdanarson T.R., Rabe K.G., Rubin J., Petersen G.M. (2008). Pancreatic neuroendocrine tumors (PNETs): Incidence, prognosis, and recent trend toward improved survival. Ann. Oncol..

[5] Rindi G., Klöppel G., Alhman H., Caplin M., Couvelard A., De Herder W.W., Erikssson B., Falchetti A., Falconi M., Komminoth P. (2006). TNM staging of foregut (neuro)endocrine tumors: A consensus proposal including a grading system. Virchows Arch..

[6] La Rosa S., Klersy C., Uccella S., Dainese L., Albarello L., Sonzogni A., Doglioni C., Capella C., Solcia E. (2009). Improved histologic and clinicopathologic criteria for prognostic evaluation of pancreatic endocrine tumors. Hum. Pathol..

[7] WHO Classification of Tumours Editorial Board (2019). Tumours of the pancreas. Digestive System Tumours, WHO Classification of Tumours.

[8] Fujimori N., Osoegawa T., Lee L., Tachibana Y., Aso A., Kubo H., Kawabe K., Igarashi H., Nakamura K., Oda Y. (2016). Efficacy of endoscopic ultrasonography and endoscopic ultrasonography-guided FNA for the diagnosis and grading of pancreatic neuroendocrine tumors. Scand. J. Gastroenterol..

[9] Falconi M., Eriksson B., Kaltsas G., Bartsch D.K., Capdevila J., Caplin M., Kos-Kudla B., Kwekkeboom D., Rindi G., Klöppel G. (2016). ENETS Consensus Guidelines Update for the Management of Patients with Functional Pancreatic Neuroendocrine Tumors and Non-Functional Pancreatic Neuroendocrine Tumors. Neuroendocrinology.

[10] Ricci C., Taffurelli G., Campana D., Ambrosini V., Pacilio C.A., Pagano N., Santini D., Brighi N., Minni F., Casadei R. (2017). Is surgery the best treatment for sporadic small (<2 cm) nonfunctioning pancreatic neuroendocrine tumours? A single centre experience. Pancreatology.

[11] Tomassetti P., Campana D., Piscitelli L., Casadei R., Santini D., Nori F., Morselli-Labate A.M., Pezzilli R., Corinaldesi R. (2005). Endocrine pancreatic tumors: Factors correlated with survival. Ann. Oncol..

[12] Fendrich V., Waldmann J., Bartsch D.K., Langer P. (2009). Surgical management of pancreatic endocrine tumors. Nat. Rev. Clin. Oncol..

[13] Jarufe N.P., Coldham C., Orug T., Mayer A.D., Mirza D.F., Buckels J.A.C., Bramhall S.R. (2005). Neuroendocrine tumours of the pancreas: Predictors of survival after surgical treatment. Dig. Surg..

[14] Panzuto F., Nasoni S., Falconi M., Corleto V.D., Capurso G., Cassetta S., Di Fonzo M., Tornatore V., Milione M., Angeletti S. (2005). Prognostic factors and survival in endocrine tumor patients: Comparison between gastrointestinal and pancreatic localization. Endocr. Relat. Cancer.

[15] Haynes A.B., Deshpande V., Ingkakul T., Vagefi P.A., Szymonifka J., Thayer S.P., Ferrone C.R., Wargo J.A., Warshaw A.L., Fernandez-del Castillo C. (2011). Implications of incidentally discovered, nonfunctioning pancreatic endocrine tumors: Short-term and long-term patient outcomes. Arch. Surg..

[16] Bettini R., Partelli S., Boninsegna L., Capelli P., Crippa S., Pederzoli P., Scarpa A., Falconi M. (2011). Tumor size correlates with malignancy in nonfunctioning pancreatic endocrine tumor. Surgery.

[17] Scarpa A., Mantovani W., Capelli P., Beghelli S., Boninsegna L., Bettini R., Panzuto F., Pederzoli P., Delle Fave G., Falconi M. (2010). Pancreatic endocrine tumors: Improved TNM staging and histopathological grading permit a clinically efficient prognostic stratification of patients. Mod. Pathol..

[18] Wente M.N., Veit J.A., Bassi C., Dervenis C., Fingerhut A., Gouma D.J., Izbicki J.R., Neoptolemos J.P., Padbury R.T., Sarr M.G. (2007). Postpancreatectomy hemorrhage (PPH): An International Study Group of Pancreatic Surgery (ISGPS) definition. Surgery.

[19] Bassi C., Marchegiani G., Dervenis C., Sarr M., Abu Hilal M., Adham M., Allen P., Andersson R., Asbun H.J., Besselink M.G. (2017). The 2016 update of the International Study Group (ISGPS) definition and grading of postoperative pancreatic fistula: 11 years after. Surgery.

[20] Gierek M., Merkel K., Ochala-Gierek G., Niemiec P., Szyluk K., Kusnierz K. (2022). Which suture to choose in hepato-pancreatic-biliary surgery? Assessment of the influence of pancreatic juice and bile on the resistance of suturing materials-In vitro research. Biomedicines.

[21] La Rosa S., Sessa F., Uccella S. (2016). Mixed neuroendocrine non-neuroendocrine neoplasms (MiNENs): Unifying the concept of a heterogeneous group of neoplasms. Endocr. Pathol..

[22] Foundation for Statistical Computing, Vienna, Austria A Language and Environment for Statistical Computing. https://www.R-project.org/.

[23] Zhang Z., Liu M., Ji S., Luo G., Xu W., Liu W., Hu Q., Sun Q., Ye Z., Qin Y. (2020). Prognostic Value and Clinical Predictors of Lymph Node Metastases in Pancreatic Neuroendocrine Tumors. Pancreas.

[24] Franko J., Feng W., Yip L., Genovese E., Moser A.J. (2010). Non-functional neuroendocrine carcinoma of the pancreas: Incidence, tumor biology, and outcomes in 2,158 patients. J. Gastrointest. Surg..

[25] Gao Y., Gao H., Wang G., Yin L., Xu W., Peng Y., Wu J., Jiang K., Miao Y. (2018). Meta-analysis of Prognostic factor of Pancreatic neuroendocrine neoplasms. Sci. Rep..

[26] Martin R.C.G., Kooby D.A., Weber S.M., Merchant N.B., Parikh A.A., Cho C.S., Ahmad S.A., Kim H.J., Hawkins W., Scoggins C.R. (2011). Analysis of 6,747 Pancreatic Neuroendocrine Tumors for a Proposed Staging System. J. Gastrointest. Surg..

[27] Bilimoria K.Y., Talamonti M.S., Tomlinson J.S., Stewart A.K., Winchester D.P., Ko C.Y., Bentrem D.J. (2008). Prognostic score predicting survival after resection of pancreatic neuroendocrine tumors: Analysis of 3851 patients. Ann. Surg..

[28] Goh B.K.P., Chow P.K.H., Tan Y.-M., Cheow P.-C., Chung Y.-F.A., Soo K.-C., Wong W.-K., Ooi L.L.P.J. (2011). Validation of five contemporary prognostication systems for primary pancreatic endocrine neoplasms: Results from a single institution experience with 61 surgically treated cases. ANZ J. Surg..

[29] Wong J., Fulp W.J., Strosberg J.R., Kvols L.K., Centeno B.A., Hodul P.J. (2014). Predictors of lymph node metastases and impact on survival in resected pancreatic neuroendocrine tumors: A single-center experience. Am. J. Surg..

[30] Sallinen V., Haglund C., Seppanen H. (2015). Outcomes of resected nonfunctional pancreatic neuroendocrine tumors: Do size and symptoms matter?. Surgery.

[31] Fathi A.H., Romanyshyn J., Barati M., Choudhury U., Chen A., Sosa J.A. (2020). Predicting Aggressive Behavior in Nonfunctional Pancreatic Neuroendocrine Tumors with Emphasis on Tumor Size Significance and Survival Trends: A Population-Based Analysis of 1787 Patients. Am. Surg..

[32] Cherenfant J., Stocker S.J., Gage M.K., Du H., Thurow T.A., Odeleye M., Schimpke S.W., Kaul K.L., Hall C.R., Lamzabi I. (2013). Predicting aggressive behavior in nonfunctioning pancreatic neuroendocrine tumors. Surgery.

[33] Hamilton N.A., Liu T.-C., Cavatiao A., Mawad K., Chen L., Strasberg S.S., Linehan D.C., Cao D., Hawkins W.G. (2012). Ki-67 predicts disease recurrence and poor prognosis in pancreatic neuroendocrine neoplasms. Surgery.

[34] Milione M., Maisonneuve P., Pellegrinelli A., Spaggiari P., Centonze G., Coppa J., Delconte G., Droz dit Busset M., Lanhazo O., Pruneri G. (2019). Ki-67 and presence of liver metastases identify different progression-risk classes in pancreatic neuroendocrine neoplasms (pNEN) undergoing resection. Eur. J. Surg. Oncol..

[35] Xu J.-Z., Wang W.-Q., Zhang S.-R., Xu H.-X., Wu C.-T., Qi Z.-H., Gao H.-L., Ni Q.-X., Liu L., Yu X.-J. (2018). Intrinsic Contact between T and N Classifications in Resected Well-Moderately Differentiated Locoregional Pancreatic Neuroendocrine Neoplasms. Ann. Surg. Oncol..

[36] Bilimoria K.Y., Bentrem D.J., Merkow R.P., Tomlinson J.S., Stewart A.K., Ko C.Y., Talamonti M.S. (2007). Application of the pancreatic adenocarcinoma staging system to pancreatic neuroendocrine tumors. J. Am. Coll. Surg..

[37] Zaidi M.Y., Lopez-Aguiar A.G., Switchenko J.M., Lipscomb J., Andreasi V., Partelli S., Gamboa A.C., Lee R.M., Poultsides G.A., Dillhoff M. (2019). A Novel Validated Recurrence Risk Score to Guide a Pragmatic Surveillance Strategy after Resection of Pancreatic Neuroendocrine Tumors: An International Study of 1006 Patients. Ann. Surg..

[38] Birnbaum D.J., Turrini O., Vigano L., Russolillo N., Autret A., Moutardier V., Capussotti L., le Treut Y.-P., Delpero J.-R., Hardwigsen J. (2015). Surgical Management of Advanced Pancreatic Neuroendocrine Tumors: Short-Term and Long-Term Results from an International Multi-institutional Study. Ann. Surg. Oncol..

[39] Genç C.G., Falconi M., Partelli S., Muffatti F., Van Eeden S., Doglioni C., Klümpen H.J., Van Eijck C.H.J., Nieveen van Dijkum E.J.M. (2018). Recurrence of pancreatic neuroendocrine tumors and survival predicted by Ki67. Ann. Surg. Oncol..

[40] Partelli S., Muffatti F., Rancoita P.M.V., Andreasi V., Balzano G., Crippa S., Doglioni C., Rubini C., Zamboni G., Falconi M. (2019). The size of well differentiated pancreatic neuroendocrine tumors correlates with Ki67 proliferative index and is not associated with age. Dig. Liver Dis..

[41] Sallinen V.J., Le Large T.Y.S., Tieftrunk E., Galeev S., Kovalenko Z., Haugvik S.-P., Antila A., Franklin O., Martinez-Moneo E., Robinson S.M. (2018). Prognosis of sporadic resected small (≤2 cm) nonfunctional pancreatic neuroendocrine tumors—A multi-institutional study. HPB.

[42] Sho S., Court C.M., Winograd P., Toste P.A., Pisegna J.R., Lewis M., Donahue T.R., Hines O.J., Reber H.A., Dawson D.W. (2019). A Prognostic Scoring System for the Prediction of Metastatic Recurrence Following Curative Resection of Pancreatic Neuroendocrine Tumors. J. Gastrointest. Surg..

[43] Hüttner F.J., Schneider L., Tarantino I., Warschkow R., Schmied B.M., Hackert T., Diener M.K., Büchler M.W., Ulrich A. (2015). Palliative resection of the primary tumor in 442 metastasized neuroendocrine tumors of the pancreas: A population-based, propensity score-matched survival analysis. Langenbecks Arch. Surg..

[44] Frilling A., Li J., Malamutmann E., Schmid K.-W., Bockisch A., Broelsch C.E. (2009). Treatment of liver metastases from neuroendocrine tumours in relation to the extent of hepatic disease. Br. J. Surg..

[45] Schurr P.G., Strate T., Rese K., Kaifi J.T., Reichelt U., Petri S., Kleinhans H., Yekebas E.F., Izbicki J.R. (2007). Aggressive surgery improves long-term survival in neuroendocrine pancreatic tumors: An institutional experience. Ann. Surg..

[46] Sarmiento J.M., Heywood G., Rubin J., Ilstrup D.M., Nagorney D.M., Que F.G. (2003). Surgical treatment of neuroendocrine metastases to the liver: A plea for resection to increase survival. J. Am. Coll. Surg..

[47] Touzios J.G., Kiely J.M., Pitt S.C., Rilling W.S., Quebbeman E.J., Wilson S.D., Pitt H.A. (2005). Neuroendocrine hepatic metastases: Does aggressive management improve survival?. Ann. Surg..

